# Reduction of death anxiety in patients with advanced cancer in short-term psychotherapy

**DOI:** 10.3389/fpsyg.2025.1491734

**Published:** 2025-04-02

**Authors:** Paula Oberth, Franziska Springer, Antje Lehmann-Laue, Anja Mehnert-Theuerkauf

**Affiliations:** Department of Medical Psychology and Medical Sociology, University Medical Center Leipzig, Leipzig, Germany

**Keywords:** psychooncology, psychooncological intervention, palliative psychologists, palliative psychological support, CALM

## Abstract

**Introduction:**

The fear of one’s own dying, death anxiety, has increasingly become the focus of research in recent years. So far, we know little about the reduction of death anxiety through psychotherapeutic interventions in patients with advanced cancer and possible predictors of this reduction. The aim of this study is to investigate the extent to which death anxiety is reduced during the course of psychotherapeutic interventions and whether specific socio-demographic, psychological or medical variables can predict such reduction over time.

**Materials and methods:**

This study is a secondary data analysis of the randomized controlled trial on the effectiveness of Managing Cancer and Living Meaningfully (CALM) Therapy, a short-term psychotherapy used to reduce depression and psychological distress in patients with advanced cancer. The active control group, a non-specific psychotherapeutic counselling intervention (SPI), showed equal effects on depression and distress and consequently both groups are investigated together in this study. Within the present study, we analyze the reduction of death anxiety from baseline to 3- and 6-months follow-up. Data were collected using validated questionnaires; death anxiety was assessed using an adapted version of the Death and Dying Distress Scale (DADDS). Predictors of the reduction of death anxiety were investigated using multiple linear regression models.

**Results:**

The sample comprised 194 patients (average age 58 years, 62% female, all with a UICC stage of III or IV). There was a significant reduction of death anxiety over time, in particular between the baseline and 3 months follow-up [t (148) = 5.26, *p* < 0.001, d = 0.43] and between the baseline and 6 months follow-up [t (120) = 5.48, *p* < 0.001, d = 0.50]. The UICC disease stage III (*p* = 0.05) as well as an elevated death anxiety score/level at baseline (*p* = 0.01) were found to be predictors for the reduction of death anxiety. No further sociodemographic and medical predictors were found within the study.

**Conclusion:**

The study results suggest that psychotherapeutic interventions could reduce death anxiety in patients with advanced cancer. A time effect cannot be excluded and further studies using a care-as-usual control group are necessary. Nevertheless, this study sheds light on the role that psychotherapeutic interventions play in reducing death anxiety and complements the use of palliative medical treatments to alleviate patients’ discomfort.

## 1 Introduction

Cancer is a disease that is associated with a high mortality rate despite constantly improving therapies. According to the global cancer statistics (globoscan), around 19.3 million people worldwide were newly diagnosed with cancer in 2020 and almost 10 million died from their illness. Forecasts suggest that the number of new cases per year could be as high as 28.4 million by 2040, which would correspond to an incidence increase of 47% (Sung et al., 2021). Those affected are often confronted with various cancer-specific stressors and fears, such as increased psychological distress (Mehnert et al., 2018; Huda et al., 2022; Pinto et al., 2019; Shin et al., 2020), clinically relevant anxiety or depression (Grassi et al., 2023; Ghamari et al., 2023; Walker et al., 2020) or the fear of a return or spread of the disease; i.e. fear of progression. Increasingly, research is also focusing on the fear of one’s own dying, death anxiety. It is defined as the experience of fear, panic, dread and other fears associated with the awareness of one’s own mortality as well as frightening thoughts about what might happen after death (Gonen et al., 2012). The prevalence rates of death anxiety in patients with advanced cancer are inconsistent in research, ranging from 32% with an at least moderate severity (Neel et al., 2015), to the estimation that at least 22–55% of the patients experience moderate levels of death anxiety (Vehling et al., 2017). A meta-analysis also showed that death anxiety was higher in Asian studies compared to European and North American studies (Soleimani et al., 2020a). However, we still know little about the predictors that contribute to or explain death anxiety and how we could reduce death anxiety. Nevertheless, we know that patients with advanced cancer are often under a great deal of psychological stress and have corresponding requirements and wishes regarding the content of psychological counselling and interventions (Oberth et al., 2024).

Some studies have highlighted possible protective factors in relation to death anxiety in patients with advanced cancer, such as religiosity (Soleimani et al., 2020b; Rybarski et al., 2023), self-esteem or resilience (Hong et al., 2022). Other studies have focused on identifying patient groups that are particularly vulnerable to death anxiety: In their meta-analysis, Soleimani et al. (2020a, 2020b) found that women experience higher levels of death anxiety than men. Other studies demonstrated that younger patients with a palliative illness have a higher risk of developing anxiety and depressive disorders (Yang et al., 2017), experience more emotional stress as well as more illness-specific intrusive thoughts than older patients (Wenzel et al., 1999). Age also appears to be a predictor of death anxiety in women, but not in men (Assari and Moghani Lankarani, 2016). It is also known from psycho-oncology that people who are married or living in partnerships require less psychosocial support, as they presumably support themselves more through their partnerships and thus experience fewer fears (Faller et al., 2016; You et al., 2022). There are also links between individual attachment experiences and death anxiety (Scheffold et al., 2018; Philipp et al., 2017; Philipp et al., 2021), demoralization and death anxiety (Philipp et al., 2017; An et al., 2018) and the severity of cancer-specific symptom burden in relation to death anxiety (Mercadante et al., 2019; Su et al., 2022; Neel et al., 2015).

As part of a randomized controlled trial, our research group investigated the extent to which the short-term psychotherapy Managing Cancer and Living Meaningfully (CALM; Mehnert et al., 2020; Scheffold et al., 2015) is superior to an active control group of a non-manualized, non-specific psychotherapeutic counselling intervention (SPI) in reducing depressive symptoms and distress in patients with advanced cancer. CALM is a manualized, semi-structured, short-term supportive-expressive psychotherapeutic intervention designed specifically for patients with advanced cancer in an individual setting, comprising 3–6 individual sessions (Rodin and Hales, 2021; Rodin et al., 2018). CALM encompasses four areas: symptom management and communication with health care providers; changes in self and relationships with others; spirituality, meaning and purpose; and preparing for the future, maintaining hope and facing mortality. Therapeutic techniques are derived from cognitive behavioral techniques and/or psychodynamic treatment, including psychoeducation and emotion regulation techniques (Rodin et al., 2018). Previous studies using CALM were also able to demonstrate, among other things, that the death anxiety experienced by patients with advanced cancer was reduced in the CALM intervention group (Rodin et al., 2018). The primary results of the study of our research group have demonstrated CALM and SPI both to be effective in improving symptoms of distress and depression, as well as death anxiety (secondary outcome). These results could be due to an insufficient treatment differentiation between CALM and SPI (Mehnert et al., 2020); hence a retrospective content analysis of the SPI interventions did not show a difference in content compared to the CALM interventions (Koranyi et al., 2020). Based on these prior results, this secondary data analysis aims to answer the following questions:

1. Does death anxiety decrease in advanced cancer patients over the course of the psychooncological interventions?
2. Which socio-demographic, medical and psychological variables predict the reduction of death anxiety over time?

## 2 Materials and methods

### 2.1 Study design and participants

The current study is a secondary data analysis of the randomized-controlled trial of Managing Cancer and Living Meaningfully (CALM; Mehnert et al., 2020; Scheffold et al., 2015), which investigated the effectiveness of the CALM intervention compared to an active control group in which a non-manualized, supportive, psycho-oncological intervention (SPI) was offered. A total of 206 out of 329 eligible patients participated in the original CALM study (61.2% female; age: M = 57.9; 84.5% UICC IV stage). The participants were either assigned to the CALM intervention group (*n* = 99) or to the SPI group (*n* = 107). A reduction in depression and anxiety as well as an improvement in the patients’ quality of life was demonstrated in both groups. However, no significant difference was found between the two study groups (Mehnert et al., 2020). Thus, in the current study, both groups were analyzed as one group. The study was conducted between April 2013 and December 2018 at the Department of Medical Psychology and Medical Sociology at the Leipzig University Hospital and at the Institute and Polyclinic for Medical Psychology at Hamburg-Eppendorf University Hospital. All participants provided written informed consent prior to study participation. Adult patients with a malignant solid tumor disease, UICC disease stage III or IV, a depression score > 9 (PHQ-9 score) or a distress score = > 5 (DT score), fluency in German and no evidence of a cognitive impairment were eligible for participation. A systematic interview was conducted with all patients to check for the following exclusion criteria: lack of interest in participation, unwillingness or inability to participate in all sessions offered, acute suicidality, communicative deficits, a score of <20 on the Short-Orientation-Memory-Concentration-Test or a score of <70 on the Karnofsky-Index (Scheffold et al., 2015; Mehnert et al., 2020). The positive votes of the ethics committees are available (Hamburg PV4435; Leipzig 143-14-14042014). The study was conducted at three measurement points: at baseline (T0), 3-month follow-up (T1) and 6-months follow-up (T2). At each measurement point, patients received a psychological interview within the respective treatment alternative and were asked to complete various validated questionnaires. For more details regarding sampling, recruitment and data assessment, see Mehnert et al., 2020.

### 2.2 Data collection tools

The socio-demographic characteristics (age, gender, partnership status) were collected using a standardized self-reporting questionnaire. In addition, all medical and disease-specific data (diagnosis, current metastases, UICC disease stage, previous psychotherapeutic experience) were taken from each patient’s medical records. The survey instruments consist of the following validated questionnaires:

#### 2.2.1 The death and dying distress scale—adapted short version (DADDS-G)

The Death and Dying Distress Scale (Lo et al., 2011; Krause et al., 2015) is a validated questionnaire that records thoughts and feelings in connection to the death and dying process in cancer patients. For example, the fear of dying, the fear of missing time or the worry of being a burden for other people are addressed. The adapted short version (DADDS-G) with 9 items (Engelmann et al., 2016) is recorded on a 5-point Likert-scale from 0 (no stress) to 4 (very severe stress). A mean value is computed, with higher values indicating greater fear of death or stress. The scale shows excellent internal consistency (Cronbach alpha = 0.91; Engelmann et al., 2016).

#### 2.2.2 The experience in close relationships inventory—adapted short version (ECR-M16-G)

The Experience in Close Relationships Inventory (ECR; Brennan Kelly et al., 1998; Ehrenthal et al., 2009) measures people’s attachment experiences in relationships and focuses on the anxious and avoidant attachment styles. The adapted short version with 16 items (Lo et al., 2009) measures the two subscales attachment anxiety and attachment avoidance on a 7-point Likert scale (1 = no agreement; 7 = agreement). A higher sum score indicates higher attachment insecurity. Both subscales show good internal consistency (attachment anxiety: Cronbach’s α = 0.84, attachment avoidance: Cronbach’s α = 0.83) (Philipp et al., 2017).

#### 2.2.3 Demoralization-scale (DS)

The Demoralization Scale questionnaire (DS; Clarke and Kissane, 2002; Kissane et al., 2001; Kissane et al., 2004) was developed to measure demoralisation in patients with advanced cancer. The questionnaire was translated into German (Mehnert et al., 2011) and uses 24 items and four subscales to measure loss of meaning and purpose, discouragement, dysphoria and sense of failure. The German translation of the questionnaire shows a high internal consistency; Cronbach’s α = 0.84 (Mehnert et al., 2011).

#### 2.2.4 Distress-thermometer (DT)

The study used the German Version of the Distress-Thermometer (DT; Mehnert et al., 2006) that measures the psychological distress of patients with cancer. With this screening method, the psychological distress of advanced cancer patients can be recorded using a visual analogue scale from 0 (‘not stressed at all’) to 10 (‘extremely stressed’). Patients indicate how stressed they had felt in the past week.

#### 2.2.5 Memorial symptom assessment scale—adapted short version (MSAS)

The Memorial Symptom Assessment Scale (MSAS; Portenoy et al., 1994) records the cancer-specific symptom burdens. The adapted and shortened Version (MSAS-SF; Chang et al., 2000) was used in the study. It measures 32 symptoms in terms of their frequency and severity and enables the calculation of subscales for physical symptoms, psychological symptoms and a global stress index. Based on the study by Scheffold et al. (2018), the four explicitly psychological items (lack of concentration, problems with sleep, sexual problems, changes in body perception) were not included in the calculations to be able to only address somatic symptom changes.

### 2.3 Statistical analysis

The data was analyzed using IBM SPSS Statistics 29 (IBM Corporation, Armonk NY). Descriptive statistics were used to calculate the sample characteristics for the entire sample. Descriptive methods and mean comparisons via t-tests (between the measurement times T0, T1 and T2) were used to address the first question, thus to determine the course of death anxiety. The effect sizes for all group comparisons were given as Cohen’s d, with effects at d = 0.20 (small effect), d = 0.50 (medium effect) and d = 0.80 (large effect) (Cohen, 2016). To address the second question, a multilevel approach was used to analyze the associated factors for the reduction of death anxiety over time (T2-T0). In separate univariate linear regression models, with the dependent variable reduction of death anxiety, relevant factors were identified. The following were examined as independent variables: sociodemographic variables (age, gender, partnership status, psychotherapeutic experience in years), medical variables (diagnosis group, UICC disease stage, metastases, cancer-specific symptom burden; MSAS) and psychological variables at T0 Baseline (attachment uncertainty; ECR-M16-G, demoralization; DS, death anxiety; DADDS-G, distress; DT). Subsequently, all relevant factors from the univariate regression models (*p* < 0.05) were integrated into a multiple linear regression model. Results of *p* < 0.05 were considered statistically significant for all analyses.

## 3 Results

### 3.1 Study sample

**Figure 1:**
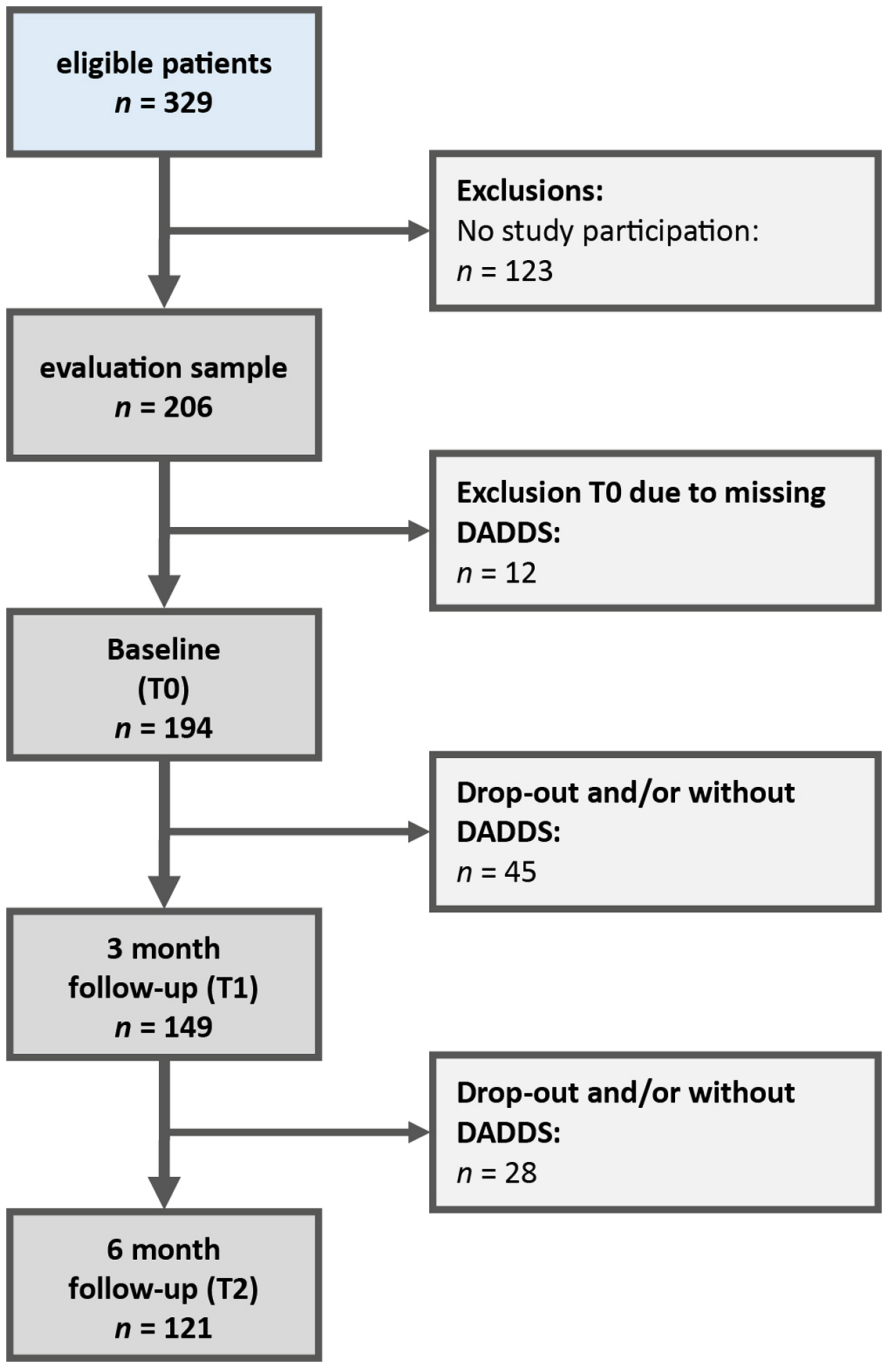
Flowchart patient recruitment.

Of 329 eligible patients, 206 patients were included in the study. 123 patients (37%) declined to participate in the study (response rate: 63%). In the current secondary data analysis, all patients who had not completed the Death and Dying Distress Scale at Baseline (T0) were also excluded (*n* = 12), as this value formed the basis for all further calculations, so that the final evaluation sample comprised *n* = 194 patients (Figure 1).

Study participants and non-participants did not differ in terms of age (study participants: 57.9 years, SD = 11.7; non-participants: 57.7 years, SD = 12.0) or gender (study participants: 61% female; non-participants: 63% female). However, non-participants were more frequently diagnosed with gastrointestinal tumors (60% vs. 40%), lung tumors (25% vs. 15%) and breast cancer (33% vs. 19%) (*p* < 0.001) compared to the study participants (cf. Mehnert et al., 2020).

There were no differences in age (study participants: 57.5 years, SD = 11.3; drop-outs: 58.6 years, SD = 12.4), death anxiety at baseline (study participants: *n* = 122, SD = 0.8; drop-outs: *n* = 72, SD = 0.8) and the UICC disease stage (study participants: 60.7%; drop-outs: 39.3%) between drop-outs and participants who completed all questionnaires. However, there were differences in gender (female drop-outs: 31%; male drop-outs: 53%, *p* = 0.002).

After 3 months (T1), 149 patients were still participating in the study (drop-out rate: 23%) and 121 after 6 months (T2) (drop-out rate: 37%). The most common reasons for drop-out were death of the patient, the deterioration in patient’s general condition, lack of interest in further participation in the study and drop-out for unknown reasons.

Patients in this study were on average 58 years old (range: 29–81 years); 62% were women. Most patients were married (59%) and had children (72%). The most common tumor entities were gastrointestinal tumors (31%) and gynecological tumors (26%). In total, 16% of the patients had a relapsed cancer diagnosis (Table 1).

**Table 1:** Sample description (*N* = 194).

		M	SD
Age (in years)		57,9	11,6
		*n*	%
Gender	Women	120	62
	Men	74	38
Partnership status	Single	28	14
	Married	115	59
	Divorced/ separated	28	14
	Widowed	23	12
Relationship	In relationship	136	70
Children	Yes	139	72
School education	Less than junior high school (<10 years)	38	20
	Junior High School (10y)	61	31
	High school (13 y)	95	49
Diagnosis/localization of cancer	Gastrointestinal tumors	60	31
	Gynecological tumors (+ breast tumors)	51	26
	Lung tumors	25	13
	Urogenital tumors	18	9
	Brain tumors	7	4
	Head–neck tumors	6	3
	Other tumors	27	14
UICC—disease Stadium	III	27	14
	IV	167	86

### 3.2 Course of death anxiety over time

**Figure 2:**
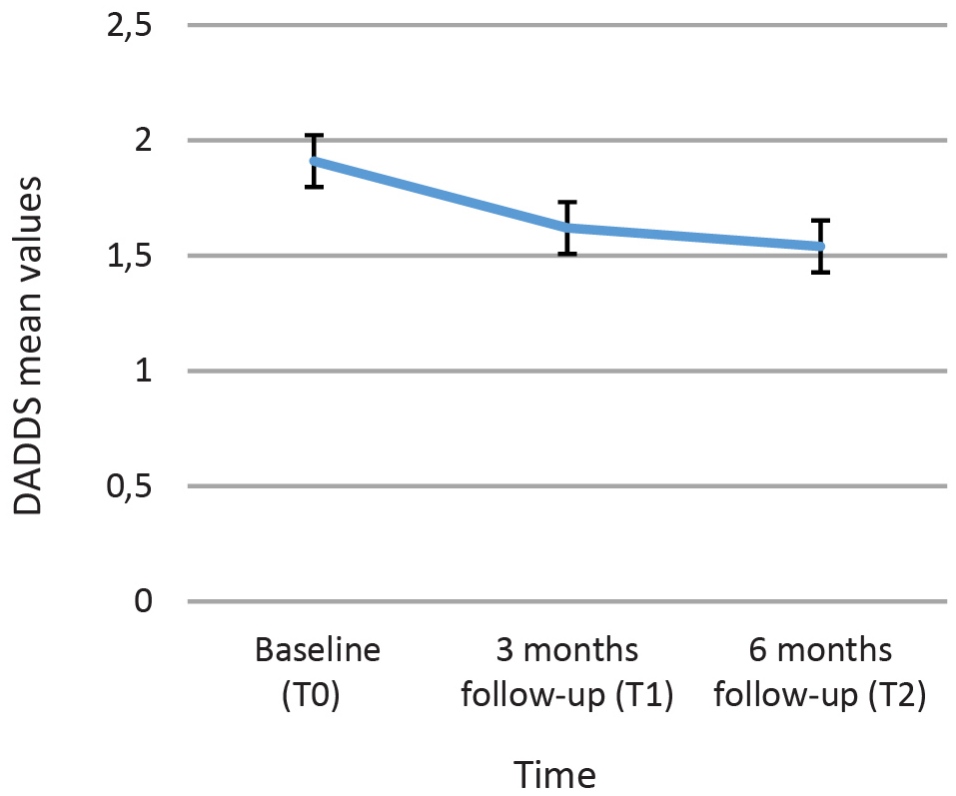
Course of the death anxiety with the mean values and 95% confidence intervals.

Mean death anxiety at Baseline (T0) was M = 1.91 (SD = 0.79), at 3 months follow-up (T1) M = 1.62 (SD = 0.81) and at 6 months follow-up (T2) M = 1.54 (SD = 0.84). Death anxiety showed a significant reduction from baseline (T0) to 3 months follow-up (T1) [t (148) = 5.26, *p* < 0.001, d = 0.43] and from baseline (T0) to 6 months follow-up (T2) [t (120) = 5.48, *p* < 0.001, d = 0.50]. There was no significant difference between T1 and T2 [t (119) = −1.43, *p* = 0.16, d = 0.13] (Figure 2).

### 3.3 Predictors for the reduction of death anxiety over time

The linear univariate regression models showed an effect on the reduction of death anxiety over time for the following variables: patient age, UICC disease stage, subscale anxiety attachment (ECR) and death anxiety (DADDS) at baseline (T0; Table 2). The following variables were not significant: gender (*p* = 0.63), partnership status (*p* = 0.48), years of psychotherapeutic experience (*p* = 0.99), current metastases (*p* = 0.74), cancer-specific symptom burden (*p* = 0.84), subjective distress (*p* = 0.62) and demoralization (*p* = 0.87).

**Table 2:** Predictors for the reduction of death anxiety over time.

	Univariate regression				Multiple regression			
	β	SE	t	*p*	β	SE	t	*p*
Age	0.26	0.01	2.88	**0.01**	0.17	0.01	1.93	0.06
UICC disease stage	0.19	0.15	2.12	**0.04**	0.17	0.15	2.02	**0.05**
Anxiety attachment (*ECR;* T0)	−0.18	0.01	−1.98	**0.05**	−0.04	0.01	−0.37	0.71
Death anxiety (*DADDS*; T0)	−0.34	0.07	−3.88	**<0.001**	−0.28	0.08	−2.798	**0.01**

Values for multiple regression; significant values are marked in bold; *N* = 118; corr. *R*^2^ = 0.151; *F* (4,113) = 6.211; *p* < 0.001.

In the multiple regression analysis, an effect for death anxiety at baseline and UICC disease stage was found. Participants with a higher death anxiety at baseline and a UICC stage III showed a greater reduction in death anxiety over time (Table 2).

## 4 Discussion

### 4.1 Summary of results

The study results show a significant reduction of death anxiety over time in patients with advanced cancer undergoing a short-term psychotherapy. Death anxiety decreased within 3 months after psychotherapy and remained low after 6 months. Many of the presumed predictors for a reduction in death anxiety could not be confirmed within the study. The UICC disease stage (III) and a high level of death anxiety at baseline could be confirmed as predictors for death anxiety, meaning that death anxiety was reduced the most in patients who were most severely affected at the beginning of the short-term therapy and had a lower UICC stage (III).

### 4.2 Discussion of the results, research perspectives and implications for practice

Death anxiety is a common problem in people with advanced cancer, which causes significant psychological stress. Therefore, it is important to identify or further explore psychotherapeutic interventions, such as CALM, to improve people’s quality of life at the end of their life, especially since alleviating discomfort is the primary goal in palliative care (Grossman et al., 2018). The results on short-term CALM psychotherapy have shown that treatment approaches tailored to the individual are effective in reducing death anxiety in patients with advanced cancer.

Within the main randomized controlled trial of Managing Cancer and Living Meaningfully (CALM; Mehnert et al., 2020; Scheffold et al., 2015), no differential effects were found between the CALM and the control group (SPI), not even with regard to death anxiety. The latter was demonstrably reduced in both active psychotherapy groups. This seems plausible, as the CALM therapy is based on psychotherapeutic interventions that have already been researched and proven to be effective for patients with advanced cancer. It is therefore conceivable that the psycho-oncologists or palliative psychologists working in the control group (SPI), with several years of clinical experience, use treatment concepts similar to those in the CALM manual, so that there were no group differences (Koranyi et al., 2020). However, it remains unclear whether this is due to a lack of treatment differentiation between the two groups or whether patient characteristics, such as gender, age or symptoms, might have an influence on therapist’s adherence and thereby on the implementation of the therapy sessions. The latter cannot be answered in the current study, as no stratified approach was followed in the study. Koranyi et al. (2020) suggested examining therapists’ adherence in future studies using CALM RCT audio recordings to see whether this was creating differential effects. At the same time, as suggested by Mehnert et al. (2020), it could be worthwhile to analyze in a future study whether there are different results when comparing CALM short-term therapy with a control group that is also undergoing a manualised supportive or supportive-expressive psycho-oncological therapy. Nevertheless, it could also not be ruled out, neither in the main study nor in the present secondary data analysis, that the effect was not a psychotherapy effect but a time effect.

The study showed that a high level of death anxiety at the beginning of treatment, as well as an UICC disease stage of III, were associated with a greater reduction in death anxiety over time. It seems plausible that patients who have only ‘tipped over’ into a palliative setting or who have also experienced strong or possibly unexpressed death anxieties benefit most from psychotherapeutic interventions and thus their death anxiety is reduced most in comparison. This is in line with the research of Grumann and Spiegel (2003), who interviewed 12 women with advanced cancer. They offered all women at least one conversation, which was sufficient for most of them. They reported that most women found it relieving to talk openly about their thoughts about death, but also about their death anxiety (Grumann and Spiegel, 2003). Accordingly, in the present study, the first psychotherapy sessions in particular were associated with the strongest reduction of death anxiety, since it was above all the act of addressing and expressing thoughts of death as well as the shared endurance that had the strongest and clarifying effect. In addition, the positive effects of the psychotherapeutic interventions were long-lasting and persisted even after 6 months.

Nevertheless, only a few of the previously assumed predictors for the reduction of death anxiety could be confirmed. Contrary to previous research findings (Soleimani et al., 2020a; Assari and Moghani Lankarani, 2016; Philipp et al., 2017; An et al., 2018; Mercadante et al., 2019; Su et al., 2022; Neel et al., 2015; You et al., 2022; Faller et al., 2016), no effects were found for gender, partnership status, degree of metastasis, demoralization, experienced distress or cancer-specific symptom burden within the patient group. Also, previous psychotherapeutic experiences of the patients had no direct influence on a reduction of the experienced death anxiety; this may be the case, as the experienced death anxiety is not a neurotic but an existential fear, for which it is difficult to prepare. In addition to the targeted predictors addressing death anxiety, it could be promising to look at possible defense mechanisms that patients use to counter existential fears, such as repression, regression or intellectualisation. Hence, it could be instructive to examine whether the choice of defense mechanisms is related to the desire for psychological conversations to counter the experienced death anxiety and could lead to a broader understanding of the interaction between psychological interventions and death anxiety.

Although attachment and age could not be confirmed as predictors for the reduction of death anxiety in the final model, both predictors were significant in the univariate calculations. Contrary to results from previous studies (Yang et al., 2017; Wenzel et al., 1999), in our study older people in particular appeared to benefit from a reduction of death anxiety over time. This seems to be in line with the considerations of Neel et al. (2015), who assume that with increasing age, more confrontations with death take place and, accordingly, more thought is given to one’s own mortality. Taking these considerations further, it would also be plausible that older and attachment-anxious people have less contact and fewer opportunities to talk about their illness-related fears and worries, as well as their death anxiety, and could therefore particularly benefit from targeted psychotherapeutic interventions or appropriately focused short-term psychotherapy.

### 4.3 Strengths and limitations of the study

The secondary data analysis of the present study is based on the randomized and controlled CALM study (Mehnert et al., 2020), which is based on a large and representative sample. One limitation of the study is that there was no control group in the study design for ethical reasons. Thus, it cannot be completely ruled out that the time elapsed between the psychotherapeutic interventions had a greater effect on the study results than the interventions themselves, even if this is very unlikely.

### 4.4 Conclusion

In summary, the study results suggest that there are only a few predictors or indicators that clearly influence the experienced death anxiety in people with advanced cancer. It appears that it is not possible to prepare for existential fears such as death anxiety, but that targeted and specialized psychotherapeutic interventions are helpful in countering the fear. With its results, the current study contributes to expanding the relevance of psychotherapeutic interventions in relation to the treatment and reduction of death anxiety in people with advanced cancer, which is urgently needed in clinical practice.

## Data Availability

The raw data supporting the conclusions of this article will be made available by the authors, without undue reservation.
